# PBAT Based Composites Reinforced with Microcrystalline Cellulose Obtained from Softwood Almond Shells

**DOI:** 10.3390/polym13162643

**Published:** 2021-08-09

**Authors:** Luigi Botta, Vincenzo Titone, Maria Chiara Mistretta, Francesco Paolo La Mantia, Aurora Modica, Maurizio Bruno, Francesco Sottile, Francesco Lopresti

**Affiliations:** 1 Department of Engineering, University of Palermo, RU INSTM, Viale delle Scienze, 90128 Palermo, Italy; vincenzo.titone@unipa.it (V.T.); mariachiara.mistretta@unipa.it (M.C.M.); francescopaolo.lamantia@unipa.it (F.P.L.M.); francesco.lopresti01@unipa.it (F.L.); 2 Centro Interdipartimentale di Ricerca “Riutilizzo Bio-Based Degli Scarti da Matrici Agroalimentari” (RIVIVE), Università degli Sudi di Palermo, 90128 Palermo, Italy; maurizio.bruno@unipa.it (M.B.); francesco.sottile@unipa.it (F.S.); 3 Dipartimento di Scienze e Tecnologie Biologiche, Chimiche e Farmaceutiche (STEBICEF), Università degli Studi di Palermo, Viale delle Scienze, 90128 Palermo, Italy; aurora.modica@unipa.it; 4 Dipartimento di Architettura, Università degli Studi di Palermo, Viale delle Scienze, 90128 Palermo, Italy

**Keywords:** biocompostable composites, natural fibers, microcrystalline cellulose, agricultural waste valorization

## Abstract

This study explores the processability, mechanical, and thermal properties of biocompostable composites based on poly (butylene adipate-co-terephthalate) (PBAT) as polymer matrix and microcrystalline cellulose (MCC) derived from softwood almond (*Prunus dulcis*) shells (as-MCC) as filler at two different weight concentration, i.e., 10 wt% and 20 wt%. The materials were processed by melt mixing and a commercial MCC (c-MCC) was used as filler comparison. The fibrillar shape of as-MCC particles was found to change the rheological behavior of PBAT, particularly at the highest concentration. The melt mixing processing allowed obtaining a uniform dispersion of both kinds of fillers, slightly reducing the L/D ratio of as-MCC fibers. The as-MCC particles led to a higher increase of the elastic modulus of PBAT if compared to the c-MCC counterparts. Both the MCC fillers caused a drastic reduction of the elongation at break, although it was higher than 120% also at the highest filler concentrations. DSC analysis revealed that both MCC fillers poorly affected the matrix crystallinity, although as-MCC induced a slight PBAT crystallinity increase from 8.8% up to 10.9% for PBAT/as-MCC 20%. Therefore, this work demonstrates the great potential of MCC particles derived from almond shells as filler for biocompostable composites fabrication.

## 1. Introduction

Considerable concerns for traditional thermoplastic polymers are increasing the demand for eco-friendly polymeric materials aiming to improve both the environment and the green economy [1]. Biocompostable composites are emerging as new eco-friendly materials for commercial and engineering applications due to their potential sustainability from an economic and ecological point of view [2]. These materials are commonly produced by combining biocompostable polymers with particles collected from natural resources [3].

A wide plethora of biocompostable polymers, such as polylactic acid [4,5], starch-based polymers [6,7], and polyhydroxyalkanoates [8,9] were proposed as suitable materials for biocompostable composites preparation. In this context, poly (butylene adipate-co-terephthalate) (PBAT) is an aliphatic/aromatic copolyester, fully biodegradable, that can be adopted for several applications, including agricultural and food-packaging [10,11].

In order to improve the final properties of this polymer, different natural particles were tested as fillers for PBAT, such as lignin [12,13], residual microalgae biomass [14], and torrefied coffee grounds [15]. The use of these materials served for high-end performing materials fabrication suitable for several applications and, at the same time, allowed the valorization of marine, industrial or agricultural wastes [12,13,14,15].

In this context, cellulose represents a potential easy-available and inexpensive source of raw material for the increasing demand for eco-friendly products [16]. The utilization of micro or nano-scaled cellulosic particles acctracts both academic and industrial interest as fillers for biocompostable composites preparation [17].

Microcrystalline cellulose (MCC), one of the cellulose derivatives, is a crystalline micrometric powder well-known for its biocompatibility, biodegradability, and high mechanical strength [18].

MCC can be extracted from various agricultural waste such as mangosteen [19], rice husk [20], cotton [21], oil palm [22], jute [23], or orange mesocarp [24]. Recently, MCC extracted from the wheat stalk (*Triticum aestivum*) was successfully used as filler for PBAT matrix, demonstrating to act as reinforcing phase for this polymer [25]. In our previous work [16], MCC particles were successfully extracted from softwood almond (*Prunus dulcis*) shells via an alkaline treatment.

In this work, aiming to investigate the potential of this green product obtained from a typical agricultural waste, the MCC particles extracted from softwood almonds were used as filler for a PBAT matrix. The PBAT-based biocompostable composites were prepared by melt mixing processing. In order to highlight the potential of the extracted MCC, the same biocompostable composites were prepared by using a commercial grade of MCC as filler for the same polymer matrix and compared each other. In particular, the materials were characterized in order to find correlations between processability, structural properties, thermal behavior, and mechanical properties of the PBAT-based biocompostable composites.

## 2. Materials and Methods

### 2.1. Materials

The polymer chosen as matrix is a film grade PBAT (ecoflex^®^ F Blend C1200, Basf, Ludwigshafen, Germany) with melt flow rate (MFR) in the range 2.7–4.9 g/10 min (190 °C, 2.16 kg), density in the range 1.25–1.27 g/cm^3^ and melting temperature in the range 110–120 °C.

Two different MCC samples were used as fillers for the preparation of biocomposites: a commercial sample (hereafter coded as c-MCC) supplied by Sigma Aldrich (St. Louis, MO, USA) (code: 435236, Lot: #MKCD7414, obtained from cotton linter [26]) and a sample obtained by alkaline treatment from softwood almond (*Prunus dulcis*) shells (hereafter coded as as-MCC). The as-MCC particles were obtained as reported in a previous work [16]. In brief, almond shells were ground and treated in an alkaline environment to remove hemicelluloses and lignin. Then, the product was filtered and washed with distilled water until neutrality. Finally, the material was bleached with NaClO solution 2.5% *w/v* at 70 °C for 1 h under mechanical stirring, filtered, and washed.

Aiming to avoid hydrolytic scission of ester bonds of PBAT during processing, PBAT and both the cellulose fillers were dried under vacuum for 4 h at 70 °C and overnight at 80 °C, respectively.

### 2.2. Processing

PBAT/c-MCC and PBAT/as-MCC composites were processed by a Brabender PLE330 (Duisburg, Germany) batch mixer. Different formulations were prepared through pre-mixing at the solid-state PBAT and 10 wt% or 20 wt% of as-MCC or c-MCC particles based on the composites weight. Then, the melt mixing process was carried out according to the following parameters: T = 170 °C, rotor speed *n* = 60 rpm for 5 min. During the process, the torque values were measured at different time points. At the end of the process, the system was immediately quenched in liquid nitrogen. 

500 µm thick samples were prepared by compression molding using a laboratory press (Carver, Wabash, IN, USA) at 70 °C for 120 s at operating at 100 bar to obtain samples for the analysis. Pure PBAT, used as control sample, was subjected to the same processes.

### 2.3. Characterization Techniques

Rheological investigations of the melts were carried out through a plate–plate rotational rheometer ARES-G2 (TA Instruments, New Castle, DE, USA) equipped with a parallel-plate geometry (25-mm diameter). Frequency sweep tests, from 0.1 to 100 rad/s, were performed at 170 °C. The PBAT-based samples for rheological tests were prepared via compression molding of the melt mixed systems in a 25-mm diameter and 1.5-mm thick stainless steel mold at the same conditions above described. Before testing, all the samples were let to dry under vacuum overnight at 70 °C.

A differential scanning calorimeter (Setaram, model DSC131 evo, Lyon, France) was used to assess the thermal characterization of the materials. The pre-weighed specimens (~5 mg) experienced heating/cooling ramps from 30 °C to 190 °C (scanning rate = 10 °C/min), under nitrogen gas atmosphere. The following Equation (1) was adopted for evaluating the degree of PBAT crystallinity (*χ*):

(1) $$\chi \;\left(\%\right)=\frac{\Delta H_{m}}{\Delta H^{0}{}_{PBAT\;}\times X_{PBAT}}\times 100$$

where ΔH_*m*_ is the melting enthalpy of the samples; X*_PBAT_* is the weight fraction of PBAT in the composite; and ΔH^0^*_PBAT_* is the 100% crystalline PBAT melting enthalpy (114 J/g) [27].

The morphology of the materials was observed through a Quanta 200 ESEM FEI (Hillsboro, OR, USA) scanning electron microscope (SEM). For the PBAT-based biocomposites, each specimen was fractured in liquid nitrogen and the fracture surface was analyzed. MCC-based filler morphology was observed via SEM before and after processing. More in detail, the fillers were extracted from the polymer matrix through a Soxhlet extractor for 48 h, using tetrahydrofuran (THF) as solvent of the polymer phase. as-MCC and c-MCC length (L), diameter (D) and shape factors (L/D) were measured using an image processing software freely available from the National Institute of Health USA (ImageJ, Bethesda, MD, USA) on the SEM micrographs of the as-received fillers and that extracted from PBAT-based composites.

Tensile mechanical measurements were assessed by using an Instron 3365 (Instron, Norwood, MA, USA) universal testing machine (UTM). The thickness of each rectangular shaped (10 × 90 mm) sample was measured before test. The specimens were fixed to the pneumatic jaws of the UTM put at an initial distance equal to 30 mm. The testing machine was equipped with a 1 kN load cell. By considering the high elongation of the tested samples, the measurements were performed at a crosshead speed equal to 1 mm/min for 1 min that automatically changed to 100 mm/min until sample fracture. The nominal stress-strain curves most representative of each sample were reported. The elastic modulus (E) was calculated as the slope of the initial linear region of the stress-strain curves. 

## 3. Results and Discussion

### 3.1. Processing and Rheological Characterization

**Figure 1:**
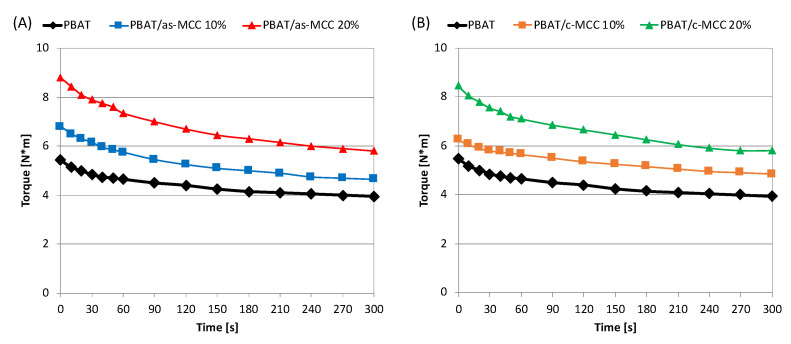
Torque as a function of time of (**A**) PBAT/as-MCC and (**B**) PBAT/c-MCC biocomposites.

Figure 1A,B shows the torque vs. time graphs recorded during melt mixing at 170 °C at 60 rpm PBAT/as-MCC PBAT and PBAT/c-MCC biocompostable composites, respectively. Although not as accurate as other rheological tests, these curves can be used for a preliminar and online analysis of the melt viscosity of the polymeric system under the chosen processing conditions [28]. After about 3–5 min, the torque curve reaches a levelling out for all the systems analyzed, thus indicating that it was achieved a complete and effective mixing of the system [29]. As clearly visible in Figure 1A,B, the torque values highlight an increasing trend as a result of the filler introduction in the polymer matrix. This result was expected since it is well known that solid particles tend to increase the viscosity of polymer melts [30]. Interestingly, the torque behavior seems to be unaffected by the kind of MCC used since the torque curve shape did not significantly change by modifying the type of MCC.

**Figure 2:**
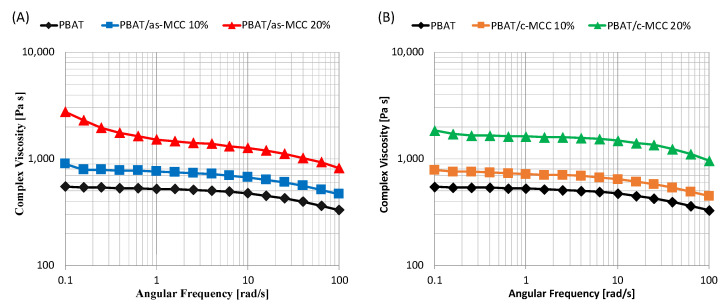
Complex viscosity as a function of frequency of (**A**) PBAT/as-MCC and (**B**) PBAT/c-MCC biocomposites.

Figure 2A,B show the complex viscosity as a function frequency of PBAT/as-MCC and PBAT/c-MCC systems, respectively. The results highlighted that the fillers significantly affected the rheological response of PBAT-based biocomposites. Coherently to the torque measurements, the viscosity of the melt increased upon increasing the filler concentration. Interestingly, Figure 2A clearly reveals that composites containing 20 wt% of as-MCC exhibited non-Newtonian behavior at lower frequencies. 

The same trend, but weaker, was also observed for PBAT/as-MCC 10% (Figure 2A), and PBAT/c-MCC 20% (Figure 2B). In fact, for these samples, the non-Newtonian behavior is sligthly more pronounced than that of the matrix. This result is in accordance with other fiber-reinforced composites in scientific literature that showed a similar behavior called shear thinning [31,32,33].

**Figure 3:**
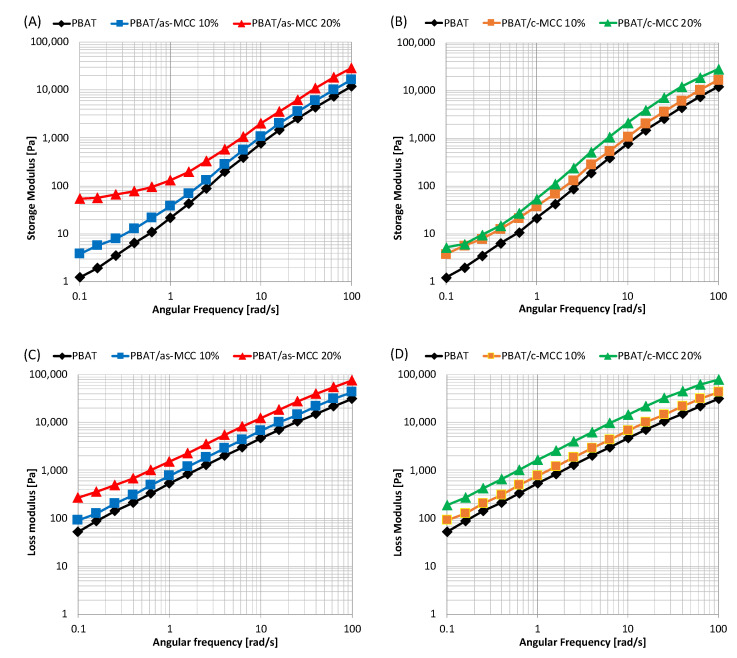
Storage modulus of (**A**) PBAT/as-MCC and (**B**) PBAT/c-MCC biocomposites; Loss modulus of (**C**) PBAT/as-MCC and (**D**) PBAT/c-MCC biocomposites.

As visible in Figure 3A–C, the increase in complex viscosity induced by the filler inclusion in PBAT is primarily caused by an increase in the storage modulus. In fact, the addition of as-MCC (Figure 3A) and c-MCC (Figure 3B) fillers to the polymer matrix increased the storage modulus of the melt that increased upon increasing the particle concentration. The corresponding increase in the loss modulus is much lower for both composites, in particular at low frequencies, as visible in Figure 3C,D. More in detail, at low frequency, the value of the storage modulus of PBAT/as-MCC 20% composite, becomes almost frequency-independent, thus indicating a transition of the viscoelastic behavior of the melted system to a solid-like behavior [34]. It is known from the scientific literature that interconnected structures of anisometric fillers in polymeric melts result in an apparent yield stress which is visible in dynamic measurements by a plateau at low frequencies of the storage modulus versus frequency curves [35,36]. This effect is definitely more pronounced in PBAT/as-MCC 20% systems although also visible in the other composites.

According to scientific literature, this result lets us reasonably assume that during the melt mixing as-MCC fibers at 20 wt% are able to form an interconnected structure by reaching a rheological percolation composition likely able to restrains the polymer relaxation process [37]. However, as shear rate increases, the 3D networks integrity is lost and the matrix contributions on the storage modulus dominate, as confirmed by the slope of the storage modulus curve in Figure 3A, very similar to that of the neat matrix [38].

The result is coherent to that of a work of Madera-Santana et al. [39] focused on PBAT/agar biocomposites. In this work, at low frequencies, the storage modulus increased upon increasing the content of agar, whereas at high frequencies, the storage modulus of PBAT/agar biocomposites is similar to that of pure PBAT.

### 3.2. Morphological Characterization

The morphology of raw as-MCC and c-MCC particles are shown in Figure 1A,B, respectively while in Table 1 the mean length, diameter and L/D ratio are summarized of the fillers.

**Figure 4:**
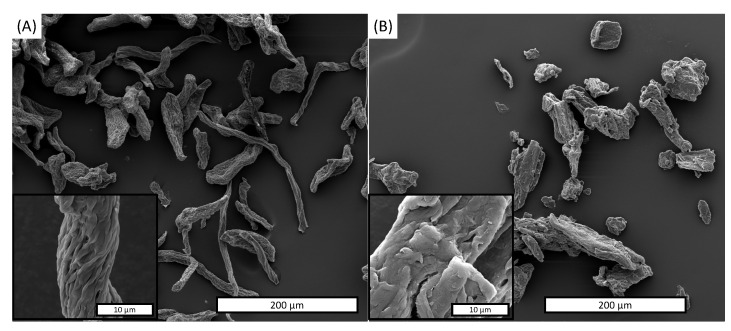
SEM image of (**A**) as-MCC and (**B**) c-MCC particles.

The micrography of as-MCC (Figure 1A) displays a microfibrillar structure with a mean diameter and length equal to 13.9 μm and 94.7 µm, respectively. As observed in our previous work, this structure is derived from the vascular bundle of the almond shell used as the cellulose source [16]. The inset at higher magnification reveals a hierarchical structure characterized by the presence of a series of parallel microbundles ascribable to crystalline cellulose entities [40,41]. SEM images of c-MCC showed in Figure 4B highlighted that the particles are in the same dimension range but less elongated than as-MCC. More in detail, for c-MCC the mean diameter and length were found to be equal to 60.9 μm and 31.6 µm, respectively. Additionally, the inset in Figure 1B denotes that c-MCC particles did not show microbundles on the surface that, in fact, appear smoother than that of as-MCC and without evidence of hierarchical structures.

The mean L/D ratio difference between as-MCC and c-MCC, equal to 6.9 and 4.3, respectively (Table 1), can furtherly explain the peculiar rheological behavior of PBAT/as-MCC 20% composite reported in Figure 1A. In fact, small and elongated fillers in a polymeric melt are able to get structured in a three-dimensional network at lower concentrations than particles characterized by lower L/D aspect ratio [42]. 

**Figure 5:**
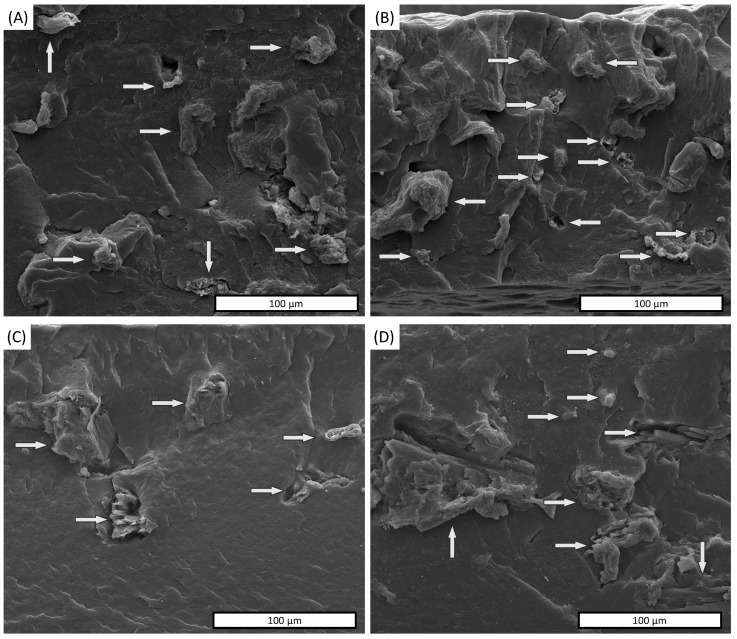
SEM images of the fractures surface of (**A**) PBAT/as-MCC 10%, (**B**) PBAT/as-MCC 20%, (**C**) PBAT/c-MCC 10%, (**D**) PBAT/c-MCC 20%.

The filler distribution was observed through SEM micrographs of the fracture surface of PBAT/as-MCC and PBAT/c-MCC composites as a function of the amount of filler loaded into the polymer matrix (Figure 5A–D). Figure 5A,B are an overview of PBAT/as-MCC 10% and PBAT/as-MCC 20% composites, respectively, showing a uniform dispersion of as-MCC in the PBAT matrix. A good filler dispersion was also observed in PBAT/c-MCC 10% and PBAT/c-MCC 20% composites shown in Figure 5C,D, respectively.

**Figure 6:**
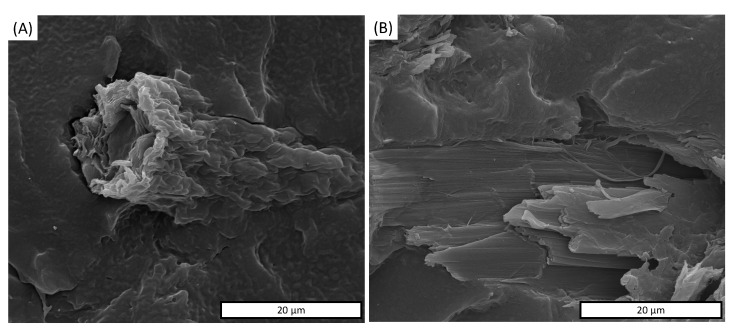
SEM images of the fractures surface of (**A**) PBAT/as-MCC 20%, (**B**) PBAT/c-MCC 20%.

SEM images also highlight that both as-MCC and c-MCC particles still maintain their original shape. The different amounts of MCC particles in the matrix are well recognizable from SEM micrographs of the cross-section of the composite that highlight an increasing number of particles for unit of area upon increasing the concentration of the filler. For all composites, the SEM overview highlighted a fiber pullout absence that usually indicates good filler-matrix adhesion. The good interfacial adhesion between MCC-based particles and PBAT was confirmed by a more detailed micrograph shown in Figure 6A,B for PBAT/as-MCC 20% and PBAT/c-MCC 20%, respectively. The as-MCC structure inside the polymer matrix showed the same fibrillar microstructure observed before inclusion in PBAT (Figure 6A). The absence of voids around the as-MCC particles indicates that there is good adhesion between the PBAT and as-MCC. A similar observation can be made for c-MCC in Figure 6B. Despite the non-polar nature of PBAT, according to Nunes et al. [43] MCC particles have abundant hydroxyl groups in their chains that may interact with the carbonyl groups of PBAT polymer chains via hydrogen bonding. Moreover, the high roughness of the MCC particles might also contribute to the improved polymer-filler adhesion [40].

Interestingly, the fractured c-MCC (Figure 6B) revealed the presence of abundant nano-structured bundles ascribable to crystalline cellulose entities not visible on the surface of the raw c-MCC particles in Figure 4B.

**Figure 7:**
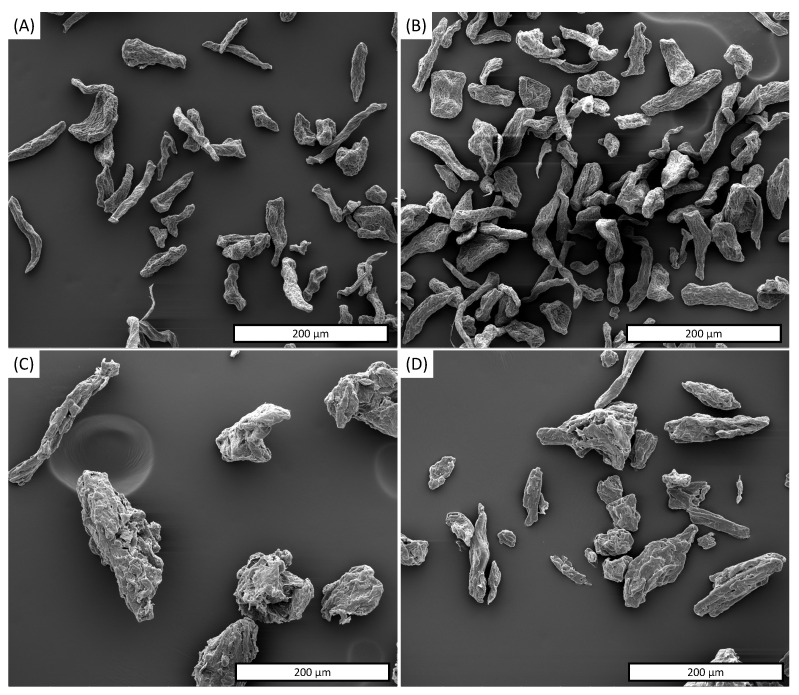
SEM images of MCC particles extracted from (**A**) PBAT/as-MCC 10%, (**B**) PBAT/as-MCC 20%, (**C**) PBAT/c-MCC 10%, (**D**) PBAT/c-MCC 20%.

In order to better understand the effect of the mixing process on the MCC-based morphology, the particles were extracted from the polymer matrix via Soxhlet extraction in THF. The morphology of the MCC-based particles extracted from the polymer matrix is reported in Figure 7A–D. More in detail, Figure 7A,B report the as-MCC particles extracted from PBAT/as-MCC 10% and 20%, respectively, while Figure 7C,D display the c-MCC particles extracted from PBAT/c-MCC 10% and 20%, respectively. In Table 1 it is possible to observe that, although the mean diameter of as-MCC was not affected by the processing, the mean length of the as-MCC fibers is lower than that of the raw filler independently from the amount loaded in PBAT.

This result can be ascribed to the shear stresses offered by the melt to the as-MCC particles during the melt mixing, partially able to break the longest particles. Figure 7C,D as well as Table 1 report that the mean length and diameter of c-MCC particles were poorly affected by the mixing process. This result can be likely ascribed to the lower L/D aspect ratio of the commercial powder (Figure 4B and Table 1). Interestingly, although the mixing processing reduced the L/D ratio of as-MCC fiber, it still doubled that of c-MCC particles (Table 1).

### 3.3. Mechanical and Thermal Properties

**Figure 8:**
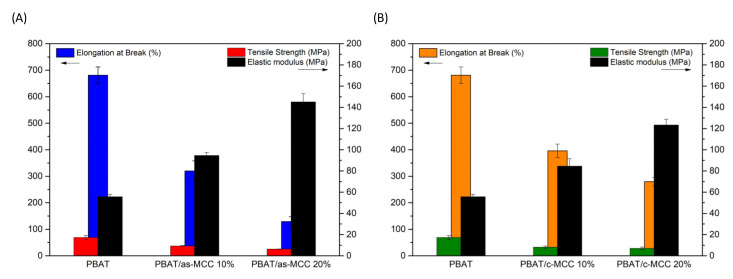
Histogram of elastic modulus, tensile strength, and deformation at break of (**A**) PBAT/as-MCC and (**B**) PBAT/c-MCC biocomposites.

Figure 8A,B summarize the tensile properties of PBAT/as-MCC and PBAT/c-MCC samples, respectively. The histogram highlights that the addition of both as-MCC and c-MCC caused a linear increase of the elastic modulus upon increasing the filler concentration. More in detail, the elastic modulus of PBAT increased from 55.5 MPa up to 145 MPa and 123.3 MPa for PBAT/as-MCC 20% and PBAT/c-MCC 20%, respectively. This result highlight that as-MCC exhibits a slightly higher reinforcing action on PBAT if compared to c-MCC.

The different elastic modulus increase observed is probably due to the as-MCC different dimension, aspect ratio, and surface roughness if compared to c-MCC particles as observed via SEM investigation (Figure 4A,B). In fact, it is well known that reducing the dimension and increasing the L/D aspect ratio of fillers usually lead to a higher reinforcing action to the polymer matrix since both these parameters reduce the concentration needed to achieve the percolation thresholds of fillers in polymeric matrices [44].

On the other hand, the presence of both as-MCC and c-MCC leads to a reduction of the tensile strength of all the composites if compared to PBAT (TS_PBAT_ = 17.3 MPa). For this parameter, the kind of MCC used as filler seemed not to affect the tensile strength that was mainly controlled by the filler concentration. More in detail, PBAT/as-MCC 10% and 20% showed a tensile strength decrease of about 46% and 64% if compared to neat PBAT, respectively. Similarly, the tensile strength decrease recorded for PBAT/c-MCC 10% and 20% was about 52% and 60%, respectively.

In addition, both the MCC-based fillers caused a reduction of the elongation at break of the polymer matrix. More precisely, the elongation at break of PBAT was 680%, while PBAT/as-MCC 10% and 20% exhibited an elongation at break about 320% and 130%, respectively. This parameter was less affected by the c-MCC particles that permitted to obtain PBAT/c-MCC 10% and 20% composites with elongation at break equal to 396% and 280%.

The decrease of the tensile strength of the composites can be associated with the premature failure of the samples, expected when rigid particles are incorporated in the polymer matrix since the filler-matrix interface, as well as the filler-voids, can act as defect in the composites despite the good matrix-filler adhesion [33].

**Figure 9:**
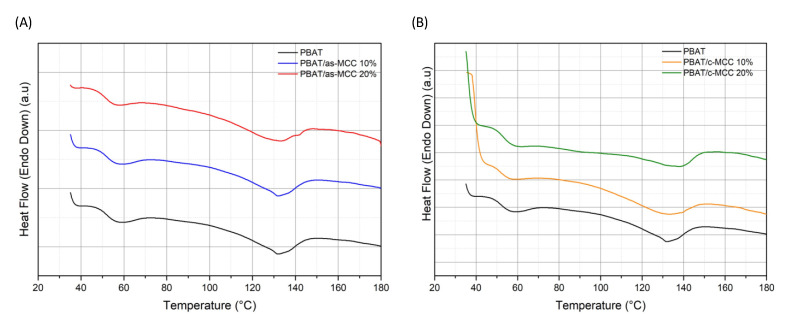
Differential scanning calorimetry (DSC) thermograms recorded during the first heating scan of (**A**) PBAT/as-MCC and (**B**) PBAT/c-MCC biocomposites.

Another parameter likely able to affect the mechanical properties of polymeric materials is crystallinity. Figure 9A,B show the DSC thermograms of PBAT/as-MCC and PBAT/c-MCC recorded during the first heating, respectively. PBAT showed the typical thermogram of an almost amorphous polymer characterized by a small endothermic peak at 125 °C related to the polymer melting phenomenon. The crystallinity values of PBAT obtained from Equation (1) and summarized in Table 2 highlight that the addition of both the fillers used in this work poorly affect this parameter. However, a trend for PBAT/as-MCC composites can be likely observed since a sligh crystallinity increase from 8.8% (for PBAT) up to 10.9% (for PBAT/as-MCC 20% biocomposites) was observed (Table 2). This result was already found in other thermoplastic polymers used as matrix for natural fibers and it was generally attributed to the nucleating action of the natural filler surface on the polymer chains [4,45,46,47]. For PBAT/as-MCC composites, the melting temperature remains unaltered by the presence of as-MCC (Table 2). Differently, when c-MCC is used as filler, a slight increase of the PBAT crystallinity was observed at the lowest c-MCC concentration and then it slightly decreased down to 8.3% for the PBAT/c-MCC 20% biocomposite (Table 2). Contextually, the same system displayed a slight increase of the melting temperature up to 130 °C (Table 2).

## 4. Conclusions

Aiming at fabricating a biocompostable composite to valorize agricultural waste, microcrystalline cellulose particles extracted from softwood almond *(Prunus dulcis)* shells were successfully used as filler for a PBAT via melt mixing. A commercial MCC was used as a comparison in order to validate the performance of the as-MCC. The rheological measurements revealed that the addition of both the fillers increased the complex viscosity of the melt upon increasing the particles loaded in the polymer matrix. A neat shear thinning behavior was recorded in PBAT/as-MCC 20% systems while it was barely present in the other systems. Furthermore, PBAT/as-MCC 20% storage modulus becomes almost frequency-independent at the lower frequency region. This result indicated a solid-like viscoelastic behavior likely ascribable to the formation of a three-dimensional network of the as-MCC particles at this concentration. This phenomenon was definitely less visible when c-MCC were used as filler, probably because of their lower L/D ratio observed via image processing of SEM micrographs. Both as-MCC and c-MCC particles were uniformly embedded in the PBAT matrix and a good matrix-filler adhesion was observed by SEM analysis. After processing, all the as-MCC particles were found to be partially broken by the shear stresses offered by the matrix during the melt mixing process. The as-MCC particles induced a higher increase of the elastic modulus if compared to the c-MCC counterparts. Due to premature failure of the samples, a drastic elongation at break reduction was observed at the highest filler content. However, the composites maintained high deformation at break higher than 120% aslo when 20 wt% of filler was loaded in the composite. DSC analysis revealed that both MCC fillers poorly affected the crystallinity of PBAT.

## Figures and Tables

**Table 1:** Length, diameter and L/D ratio of as-MCC and c-MCC raw and extracted from PBAT-based composites.

Sample	Length [µm]	Diameter [µm]	L/D
as-MCC raw	94.7 ± 19.8	13.9 ± 3.3	6.9 ± 1.8
c-MCC raw	60.9 ± 23.1	31.6 ± 8.4	2.0 ± 0.8
as-MCC from PBAT/as-MCC 10%	61.8 ± 12.1	14.2 ± 2.5	4.3 ± 1.2
as-MCC from PBAT/as-MCC 20%	57.7 ± 15.9	14.1 ± 2.1	4.1 ± 1.1
c-MCC from PBAT/c-MCC 10%	57.1 ± 18.4	29.9 ± 7.3	1.9 ± 0.8
c-MCC from PBAT/c-MCC 20%	52.6 ± 15.4	28.5 ± 7.1	1.8 ± 0.8

**Table 2:** Thermal properties collected during the second heating scan for all investigated systems.

	ΔH [j/g]	χ [%]	Tm [°C]
PBAT	10.06	8.8	125
PBAT/as-MCC 10%	10.37	9.1	125
PBAT/as-MCC 20%	12.38	10.9	126
PBAT/c-MCC 10%	11.17	9.8	126
PBAT/c-MCC 20%	9.51	8.3	128

## Data Availability

The data presented in this study are available on request from the corresponding author.
